# The impact of lymphedema severity on shoulder joint function and muscle activation patterns in breast cancer survivors: a cross-sectional study

**DOI:** 10.1007/s00520-024-09044-7

**Published:** 2024-12-17

**Authors:** Mahmoud Hamada Mohamed, Rafik E. Radwan, Mohamed M. ElMeligie, Abdelrazak Ahmed, Hend R. Sakr, Mahmoud ElShazly

**Affiliations:** 1 Department of Physical Therapy for Surgery, Faculty of Physical Therapy Ahram Canadian University, 4 Industrial Zone, Banks Complex, 6th of October City, Giza Egypt; 2 Department of Biomechanics, Faculty of Physical Therapy, Cairo University, Cairo, Egypt; 3 Department of Basic Sciences, Faculty of Physical Therapy, Ahram Canadian University, Giza, Egypt; 4 Department of Physical Therapy for Neuromuscular Disorders and Its Surgery, Faculty of Physical Therapy, South Valley University, Qena, Egypt; 5 Lecturer of Physical Therapy, Department of Women’s Health, Faculty of Physical Therapy, Badr University, Badr, Egypt; 6 Department of Physical Therapy for Surgery, Faculty of Physical Therapy, South Valley University, Qena, Egypt

**Keywords:** Lymphedema, Shoulder, Range of motion, Strength, Electromyography, Rehabilitation

## Abstract

**Purpose:**

This study aimed to investigate the impacts of breast cancer-related lymphedema (BCRL) severity on shoulder function including range of motion, strength, muscle activation patterns, and patient-reported disability.

**Methods:**

A cross-sectional, observational study design was utilized. Seventy-five women with unilateral BCRL were recruited and categorized into mild, moderate, and severe groups based on limb swelling severity. Outcomes included shoulder range of motion, isometric strength, Disabilities of Arm Shoulder and Hand (DASH) scores for disability, and surface electromyography (EMG) of shoulder muscles. Data were analyzed using parametric and nonparametric tests.

**Results:**

Increasing lymphedema severity was associated with progressive declines in shoulder mobility, strength, and function. Severe cases showed markedly reduced shoulder flexion, abduction, rotation, and extension range of motion along with decreased isometric flexor and abductor strength versus mild cases (*p* < 0.001). Higher pain levels (*p* < 0.001) and DASH disability scores (*p* < 0.001) were noted in severe BCRL. Surface EMG revealed impaired activation patterns including reduced amplitudes (*p* < 0.001) and delayed onsets (*p* < 0.001) in the deltoids, rotator cuff, and scapular muscles with greater impairment.

**Conclusions:**

Advancing BCRL severity was associated with substantial declines in shoulder mobility, strength, neuromuscular activation, pain threshold, and upper limb functionality. These quantitative results demonstrate impaired shoulder joint control underlying disability in arm elevation and daily tasks. The progressive nature of these deficits highlights the relationship between lymphedema severity and shoulder dysfunction in breast cancer survivors.

**Supplementary Information:**

The online version contains supplementary material available at 10.1007/s00520-024-09044-7.

## Introduction

Breast cancer is the second leading cause of global cancer incidence in 2022, with 2.3 million new cases (11.6% of all cancers) [1, 2]. It is the most common cancer and leading cause of cancer deaths among women worldwide, representing one in four cancer cases and one in six cancer deaths in women [2]. Incidence rates are higher in transitioned versus transitioning countries (54.1 vs 30.8 per 100,000), while mortality rates show an inverse pattern (11.3 vs 15.3 per 100,000), reflecting disparities in detection and treatment [2].

Breast cancer treatments, including lymph node dissection and radiation, can cause secondary upper extremity lymphedema termed breast cancer-related lymphedema (BCRL) [3]. As defined by the International Society of Lymphology, lymphedema is an external (and/or internal) manifestation of lymphatic system insufficiency and deranged lymph transport [4]. This lymphatic insufficiency affects up to 50% of patients receiving nodal surgery, radiation, and taxane chemotherapy, though rates are lower with conservative treatments like sentinel node biopsy [4].

This chronic condition impairs quality of life, with BCRL survivors showing 20–27 point reductions across physical, mental, and social domains according to a large study of breast cancer survivors up to 10 years post-treatment [5]. BCRL survivors face 122% higher monthly costs ($355 vs $160) 12 years post-diagnosis, with increased co-pays (131%), medications (46%), and out-of-pocket expenses (167%) [6].

BCRL manifests as upper limb swelling with associated symptoms including atrophic skin changes, pain, heaviness, decreased mobility, and recurrent cellulitis [7]. It causes significant alterations in shoulder biomechanics, particularly decreased scapular upward rotation and posterior tilt, leading to impaired upper extremity movement and function [8]. Upper limb dysfunction affects up to 82% of breast cancer survivors, limiting daily activities like reaching and self-care, with significant impact on quality of life [9]. Reduced mobility and strength worsen lymphedema and correlate with limb swelling [10].

The relationship between BCRL severity and upper-limb disability remains debated. While Ramirez-Parada et al. [11] found no association between these factors (*r* = 0.23, *p* = 0.21), Hayes et al. [12] demonstrated that early upper-body symptoms predict BCRL development (OR > 1.27, *p* < 0.05), with two-thirds of survivors reporting multiple symptoms. These contradictory findings highlight the need to better understand how BCRL and upper-limb dysfunction collectively impact survivors.

Recent evidence indicates breast cancer survivors show altered shoulder muscle coordination, with increased upper trapezius/deltoid activity and delayed activation of stabilizing muscles during arm movements [13]. Understanding how BCRL severity affects shoulder function is crucial for developing targeted rehabilitation protocols.

The purpose of this study is to investigate the relationship between lymphedema severity and shoulder joint mobility, strength, muscle activation characteristics, and self-reported upper extremity function in women with BCRL.

## Methods

### Study design

This study utilizes a cross-sectional, observational design. This study is reported in accordance with the STROBE (Strengthening the Reporting of Observational Studies in Epidemiology) guidelines [14].

### Setting

Participants will be recruited through referrals from oncologists at various hospitals in Cairo, Egypt. Referred patients will be screened for eligibility at the outpatient clinic of the Faculty of Physical Therapy at Ahram Canadian University in Giza.

Data collection was conducted at the Biomechanics and Electromyography Lab, Faculty of Physical Therapy, Ahram Canadian University, Egypt. This academic facility is staffed by licensed physical therapists and biomechanics researchers, with complete instrumentation for all study procedures.

### Participants

Seventy-five women aged 18 years or older with a diagnosis of unilateral BCRL affecting one arm for at least 3 months were included. The diagnosis was confirmed independently by the participant’s oncologist based on clinical assessment showing unilateral limb swelling and dysfunction along with medical history of breast cancer treatment [15]. Diagnostic imaging using lymphoscintigraphy was utilized to visualize lymphatic system impairment and aid diagnosis confirmation [16].

Lymphedema staging followed the International Society of Lymphology classification system [4] using multiple objective measures. Limb volume was assessed via water displacement volumetry following standardized protocols with temperature-controlled water (20–25 °C) and consistent immersion points [17]. Circumferential measurements were taken at 4-cm intervals from the ulnar styloid process to the axilla [18]. Tissue dielectric constant was measured using a MoistureMeter D Compact (Delfin Technologies, Kuopio, Finland) at standardized anatomical points [19]. Pitting edema was assessed using the standard four-point scale (0 = none to 3 = severe) with 15-s digital pressure [20].

Participants were categorized into three groups corresponding to ISL stages [4]: Stage 1 (mild/reversible edema, < 20% volume difference), stage 2 (moderate with fibrosis, 20–40% volume difference), and stage 3 (severe with tissue changes, > 40% volume difference). Active cancer was excluded through recent imaging, blood markers, and oncologist verification [21].

Exclusion criteria were as follows: (1) presence of adhesive capsulitis, rotator cuff tear, or other diagnosed shoulder pathology affecting range of motion or function identified during clinical examination, (2) previous upper extremity surgery, (3) physical inability to perform the required movements and assessments.

### Ethical considerations

The Research Ethics Committee, Faculty of Physical Therapy, South Valley University, Egypt, Protocol ID: P.T-SUR-08/2023-517 in accordance with the Declaration of Helsinki. The study was registered prospectively on ClinicalTrials.gov (NCT05934695) prior to recruitment. Written informed consent was obtained from all participants, and they were notified of their right to withdraw at any time without penalty.

### Outcomes

#### Shoulder range of motion

Shoulder flexion, abduction, external rotation, and extension ROM were measured using a digital inclinometer (Baseline Evaluation Instruments, Fabrication Enterprises Inc., USA) which has excellent inter-rater reliability (ICC 0.75–0.89) for shoulder motions [22]. Participants were positioned supine with standardized stabilization according to established protocols [23]. ROM was measured in degrees to the nearest 1° increment.

#### Shoulder strength

Isometric shoulder flexor and abductor strength were assessed using a Commander Echo Muscle Tester handheld dynamometer (JTECH Medical, Midvale, USA). Mean force in Newtons (N) was calculated from 3 maximal contractions, with 30-s rest between trials. The dynamometer has excellent inter-rater reliability (ICC 0.91–0.93) for shoulder strength assessment [24].

#### DASH questionnaire

The 30-item Disabilities of the Arm, Shoulder and Hand (DASH) questionnaire was administered to evaluate participant-reported upper extremity disability [25]. Responses are scored on a five-point Likert scale from 1 (no difficulty) to 5 (unable to perform) and used to calculate a score out of 100. The DASH demonstrates strong internal consistency (Cronbach’s α = 0.96) and test–retest reliability (ICC 0.90). [26]

#### Muscle activation patterns

Surface EMG (BIOPAC MP150, Biopac Systems Inc., USA) was used to evaluate activation patterns of shoulder musculature. Maximum voluntary contractions (MVCs) were obtained for normalization. Onset, offset, and duration were calculated during shoulder elevation. EMG has excellent reliability (ICC 0.91–0.99) for assessing shoulder dynamics [27].

### Procedure

#### Shoulder range of motion

Participants were positioned supine with the shoulder in 0° flexion/abduction and elbow extended. The scapula was stabilized by the examiner to isolate glenohumeral motion [28]. Participants performed gentle range of motion warmup exercises prior to measurements [29].

The examiner then passively moved the arm through full flexion, abduction, external rotation, and extension. Overpressure was applied at end range to ensure capsular end feel. If distal lymphedema affected inclinometer placement, it was relocated proximally to the mid-shaft of the humerus while verifying alignment via visual inspection and palpation [30]. For external/internal rotation measurements, alternate bony landmarks were used if forearm swelling was present.

Range of motion was measured using a baseline digital inclinometer accurate to 1° increments. Each ROM test was performed three times and the mean score calculated. Standardized stabilization and ROM measurement procedures followed established guidelines [31].

#### Shoulder muscle strength

Isometric shoulder strength was assessed using a Commander Echo digital handheld dynamometer (JTECH Medical, USA). This device demonstrates excellent inter-rater reliability (ICC 0.95–0.97) for upper limb strength testing [24]. Participants were seated upright in a standard armless chair, with hips and knees at 90° flexion and feet flat on the floor. The examiner stood facing the participant to provide stabilization at the elbow and verbal encouragement.

For shoulder flexion, the participant’s arm was positioned in 0° flexion/abduction with the elbow flexed to 90° and forearm neutral. The dynamometer was placed proximal to the ulnar styloid process on the dorsal surface. To test abduction strength, the arm was abducted to 90° in the plane of the scapula with the elbow extended and forearm pronated. The device was placed proximal to the ulnar styloid on the lateral forearm [32]. Proper stabilization was used to isolate glenohumeral motion.

Participants performed three maximal voluntary isometric contractions sustained for 5 s, with 30-s rest between trials. If lymphedema precluded wrist placement, the dynamometer was relocated proximally along the forearm or upper arm, in line with muscle force direction. Pilot testing in those with lymphedema verified high inter-rater reliability (ICC 0.88–0.92) using this modified technique. [33]

The mean value of the three peak force (N) measurements was calculated for analysis. Additional stabilization or body mass index normalization was applied as warranted clinically. All procedures aligned with published guidelines. [34]

#### DASH questionnaire

The DASH contains 30 items evaluating difficulty performing upper extremity activities over the preceding week. Specific tasks cover a range of daily functions including opening jars, yardwork, shopping, personal care, recreational pursuits, and occupational tasks requiring arm use. [25]

Participants independently completed the DASH questionnaire in a private room to maintain privacy. Standardized instructions were provided verbally and in written form prior to administration. The examiner remained available during completion to answer questions. Responses were self-reported on a five-point Likert scale ranging from no difficulty (1) to unable to perform (5).

Per established scoring guidelines, at least 27 of the 30 items had to be completed to calculate a valid DASH disability score [35]. The assigned values for all completed responses were summed then averaged, transformed into a score out of 100. Higher scores indicate greater disability.

#### EMG activity

Surface electromyography (EMG) was used to evaluate bilateral shoulder muscle activation patterns during elevation. Silver-silver chloride bipolar electrodes with a 10-mm diameter circular conductive area and 20-mm inter-electrode distance.

Electrode placements were determined using anatomical landmark palpation on each participant to establish optimal sites following published guidelines. For the anterior, middle, and posterior fibers of the deltoid, electrodes were positioned obliquely 2 fingerbreadths distal to the clavicular midpoint, lateral to the acromion avoiding clavicular overlap, and 2 fingerbreadths posterior to the lateral deltoid border, respectively. [36] To capture both sternal and clavicular portions of pectoralis major, electrodes were oriented near the axillary fold aligned medial to lateral and at an oblique angle below the clavicle [36].

Electrode sites were marked, digitally photographed, and re-checked across sessions to ensure consistent placement within and between participants. Minimal inter-electrode spacing and proper orientation parallel to muscle fibers optimized selectivity for the targeted shoulder muscles.

Once quality EMG signals were obtained, raw data from three full dynamic trials underwent standardized processing and analysis. Signals were bandpass filtered (20–450 Hz) to isolate muscle frequencies and improve signal-to-noise ratio [37]. Data were then full-wave rectified to extract the signal envelope and lowpass filtered (3 Hz) to create a linear envelope. [38]

Muscle onsets and offsets were determined using a threshold-based algorithm set at ± 2 SD above resting voltage, customized to each muscle’s inherent noise [39]. Onset was the first point activity rose above and remained above the threshold for ≥ 25 ms. Offset was the first point it fell and remained below the threshold for ≥ 25 ms [40].

Primary variables included activation amplitude normalized to %MVIC from 30 to 120° elevation and timing of onsets/offsets relative to anterior deltoid. Mean data across the three trials were reported. Trials were excluded if synchronization or artifact issues were visibly apparent. Excellent within-session test–retest reliability was noted for amplitude (ICC 0.92) and timing (ICC 0.89) variables.

Excel was used to process and analyze the EMG data. The raw EMG signals were imported into Excel, and the following steps were performed:

1. Bandpass filtering: Excel’s Fourier analysis functions were used to apply a bandpass filter (20–450 Hz) to isolate muscle frequencies and improve signal-to-noise ratio [41].
2. Full-wave rectification and linear envelope: Excel’s ABS function was used to full-wave rectify the data, and a lowpass filter (3 Hz) was applied using Excel’s moving average function to create a linear envelope [42].
3. Muscle onset and offset detection: A custom Excel macro was created to detect muscle onsets and offsets using a threshold-based algorithm set at ± 2 SD above resting voltage. The macro identified the first point activity rose above and remained above the threshold for ≥ 25 ms (onset) and the first point it fell and remained below the threshold for ≥ 25 ms (offset) [43].
4. Normalization and data analysis: EMG amplitude data were normalized to %MVIC using Excel’s arithmetic functions. Timing of onsets/offsets relative to anterior deltoid was calculated using Excel’s subtraction and averaging functions. Mean data across the three trials were reported, and trials with synchronization or artifact issues were excluded based on visual inspection [44].

Excel’s built-in statistical functions, such as AVERAGE, STDEV, and ICC, were used to calculate the primary variables and assess within-session test–retest reliability for amplitude (ICC 0.92) and timing (ICC 0.89) variables [45].

#### Sample size calculation

A priori power analysis using G*Power software (version 3.1.9.7) was used to calculate the minimum sample size required for this study. The output shows that a total sample size of 66 participants (22 per group) is required to detect a large effect size (*f* = 0.4) with 80% power and a significance level of 0.05. Although the minimum required sample size was 66 participants (22 per group), we decided to recruit 75 participants to increase power and for feasibility considerations. In conclusion, a sample size of 75 participants (25 per group) was recruited for this study.

## Results

### Patient demographics

A total of 75 women with unilateral breast cancer-related lymphedema (BCRL) were included in this study. The participants were categorized into mild (*n* = 25), moderate (*n* = 25), and severe (*n* = 25) groups based on the severity of their lymphedema. The mean age of the participants was 57.97 ± 8.13 years, with a significant difference between the groups (*p* = 0.012). The severe group had a significantly higher mean weight (93.52 ± 10.871 kg) and BMI (34.2 ± 2.6 kg/m^2^) compared to the mild and moderate groups (*p* < 0.001). No significant differences were observed in height (*p* = 0.123) or time since treatment (*p* = 0.453) between the groups. The distribution of treatment types was similar across the groups (*p* > 0.99) (Table 1).

**Table 1:** Patient demographics

General characteristics	Total (*n* = 75)	Mild (*n* = 25)	Moderate (*n* = 25)	Severe (*n* = 25)	*p* value
Age	57.97 ± 8.13	54.44 ± 7.405	58.32 ± 7.888	61.16 ± 7.946	0.012
Weight (kg)	82.59 ± 13.161	73.2 ± 10.132	81.04 ± 9.719	93.52 ± 10.871	< 0.001
Height (cm)	163.71 ± 5.82	162.92 ± 5.627	162.56 ± 5.561	165.64 ± 6.006	0.123
BMI (kg/m2)	30.4 (5)	26.3 (3.9)	30.4 (3)	34.2 (2.6)	< 0.001
Time since treatment (months)	18 (18)	17 (18)	18 (18)	20 (17)	0.453
Type of treatment					> 0.99
Lumpectomy	(5, 6.7)	(1, 20)	(2, 40)	(2, 40)	
Lumpectomy, chemotherapy	(11, 14.7)	(3, 27.3)	(4, 36.4)	(4, 36.4)	
Lumpectomy, chemotherapy, radiation	(13, 17.3)	(5, 38.5)	(4, 30.8)	(4, 30.8)	
Lumpectomy, radiation	(10, 13.3)	(4, 40)	(3, 30)	(3, 30)	
Mastectomy	(12, 16)	(4, 33.3)	(4, 33.3)	(4, 33.3)	
Mastectomy, chemotherapy	(7, 9.3)	(3, 42.9)	(2, 28.6)	(2, 28.6)	
Mastectomy, chemotherapy, radiation	(8, 10.7)	(2, 25)	(3, 37.5)	(3, 37.5)	
Mastectomy, radiation	(9, 12)	(3, 33.3)	(3, 33.3)	(3, 33.3)	

Values are presented as the mean ± SD, median (IQR), or (number, %)

### Pain, disability, and shoulder function

Increasing lymphedema severity was associated with significantly higher pain levels (*p* < 0.001) and DASH disability scores (*p* < 0.001). The severe group had significantly reduced shoulder range of motion (ROM) compared to the mild and moderate groups, including shoulder flexion (*p* < 0.001), abduction (*p* < 0.001), extension (*p* < 0.001), and external rotation (*p* < 0.001). Shoulder flexor and abductor strength were also significantly lower in the severe group (*p* < 0.001) (Table 2). Post hoc tests revealed significant differences between all pairwise comparisons for shoulder ROM and strength (*p* < 0.001) (Supplementary file).

**Table 2:** Comparison of Pain, DASH scores, Shoulder ROM, and EMG results across different severity levels in individuals with lymphatic edema

Variable		Mild (*n* = 25)	Moderate (*n* = 25)	Severe (*n* = 25)	*p* value
Pain (0–10 scale)		4 (2)	5 (2)	7 (2)	< 0.001
DASH scores		17.8 ± 10.603	32 ± 14.376	64.92 ± 10.214	< 0.001
Shoulder ROM	Shoulder flexion ROM (degrees)	151 (27)	118 (26)	73 (31)	< 0.001
	Shoulder abduction ROM (degrees)	144 (29)	114 (25)	70 (29)	< 0.001
	Shoulder extension ROM (degrees)	51 (10)	39 (15)	22 (9)	< 0.001
	Shoulder external rotation ROM (degrees)	76 (18)	46 (20)	33 (14)	< 0.001
	Shoulder flexor strength (N)	172 (39)	83 (26)	31 (14)	< 0.001
	Shoulder abductor strength (N)	155 (36)	98 (30)	28 (13)	< 0.001
Anterior deltoid EMG	Anterior deltoid amplitude (%MVC)	42.76 ± 2.891	32.32 ± 3.913	14.88 ± 5.819	< 0.001
	Anterior deltoid duration (ms)	970 (120)	935 (130)	890 (90)	0.010
	Anterior deltoid MVC (μV)	1200 (150)	1200 (150)	1200 (200)	0.655
	Anterior deltoid offset (ms)	950 (110)	940 (120)	905 (95)	0.182
	Anterior deltoid onset (ms)	−20 (10)	10 (10)	10 (15)	< 0.001
Middle deltoid EMG	Middle deltoid amplitude (%MVC)	42.528 ± 1.739	34.88 ± 3.504	23.04 ± 3.494	< 0.001
	Middle deltoid duration (ms)	950 (90)	910 (90)	870 (80)	< 0.001
	Middle deltoid MVC (μV)	1200 (200)	1200 (150)	1200 (150)	0.834
	Middle deltoid offset (ms)	940 (95)	930 (85)	875 (90)	< 0.001
	Middle deltoid onset (ms)	− 10 (10)	10 (10)	10 (10)	< 0.001
Posterior deltoid EMG	Posterior deltoid amplitude (%MVC)	37.96 ± 1.767	33.24 ± 3.887	13.16 ± 5.942	< 0.001
	Posterior deltoid duration (ms)	850 (100)	750 (50)	735 (37)	< 0.001
	Posterior deltoid MVC (μV)	1050 (150)	1050 (150)	1050 (150)	0.943
	Posterior deltoid offset (ms)	810 (95)	760 (55)	750 (33)	< 0.001
	Posterior deltoid onset (ms)	− 40 (10)	10 (10)	10 (10)	< 0.001
Biceps EMG	Biceps amplitude (%MVC)	32.96 ± 4.587	23.6 ± 4.031	13.4 ± 3.916	< 0.001
	Biceps duration (ms)	600 (25)	460 (40)	265 (40)	< 0.001
	Biceps MVC (μV)	779.2 ± 46.719	626 ± 41.533	432.8 ± 51.114	< 0.001
	Biceps offset (ms)	610 (40)	480 (50)	320 (50)	< 0.001
	Biceps onset (ms)	26.2 ± 19.164	37 ± 21.164	72.6 ± 35.447	< 0.001
Pectoralis major EMG	Pectoralis major amplitude (%MVC)	27.72 ± 4.852	24.96 ± 4.987	22.56 ± 4.727	0.002
	Pectoralis major duration (ms)	697.8 ± 98.129	603.6 ± 94.863	426.4 ± 97.399	< 0.001
	Pectoralis major MVC (μV)	898.8 ± 112.150	797.2 ± 106.478	597.2 ± 106.478	< 0.001
	Pectoralis major offset (ms)	722.2 ± 91.265	638.8 ± 85.626	477.6 ± 84.608	< 0.001
	Pectoralis major onset (ms)	24.4 ± 16.222	35.2 ± 21.237	51.2 ± 28.624	< 0.001

Values are presented as the mean ± SD, or median (IQR) for normally and non-normally distributed variables respectively

### Muscle activation patterns

Surface EMG analysis revealed significant differences in muscle activation patterns across the severity groups. The severe group demonstrated significantly lower EMG amplitudes (%MVC) in the anterior deltoid (*p* < 0.001), middle deltoid (*p* < 0.001), posterior deltoid (*p* < 0.001), biceps (*p* < 0.001), and pectoralis major (*p* = 0.002) compared to the mild and moderate groups. The severe group also showed significantly delayed onset times in the anterior deltoid (*p* < 0.001), middle deltoid (*p* < 0.001), posterior deltoid (*p* < 0.001), biceps (*p* < 0.001), and pectoralis major (*p* < 0.001). Muscle activation duration was significantly shorter in the severe group for the middle deltoid (*p* < 0.001), posterior deltoid (*p* < 0.001), biceps (*p* < 0.001), and pectoralis major (*p* < 0.001) (Table 2). Post hoc tests revealed significant differences between the mild and severe groups for most EMG parameters (*p* < 0.001) (Supplementary file).

### Correlations between lymphedema severity and outcomes

Lymphedema severity showed significant positive correlations with age (*r* = 0.327, *p* = 0.004), weight (*r* = 0.631, *p* < 0.001), BMI (*r* = 0.774, *p* < 0.001), pain (*r* = 0.642, *p* < 0.001), and DASH scores (*r* = 0.831, *p* < 0.001). Significant negative correlations were observed between lymphedema severity and shoulder ROM (*r* = − 0.857 to − 0.918, *p* < 0.001), strength (*r* = − 0.943, *p* < 0.001), and EMG amplitudes (*r* = − 0.388 to − 0.942, *p* < 0.001). Lymphedema severity was positively correlated with delayed muscle onset times (*r* = 0.411 to 0.742, *p* < 0.001) and negatively correlated with muscle activation duration (*r* = − 0.352 to − 0.943, *p* < 0.001) (Table 3).

**Table 3:** Correlation matrix between severity of lymphatic edema and measured outcomes

Variables	*r* values	*p* values
Age	0.327	0.004
Height (cm)	0.166	0.155
Weight (kg)	0.631	< 0.001
BMI (kg/m^2^)	0.774	< 0.001
Time since treatment (months)	0.146	0.211
Biceps MVC (μV)	− 0.942	< 0.001
Biceps amplitude (%MVC)	− 0.9	< 0.001
Biceps onset (ms)	0.556	< 0.001
Biceps offset (ms)	− 0.943	< 0.001
Biceps duration (ms)	− 0.943	< 0.001
Anterior deltoid MVC (μV)	− 0.093	0.428
Anterior deltoid amplitude (%MVC)	− 0.938	< 0.001
Anterior deltoid onset (ms)	0.742	< 0.001
Anterior deltoid offset (ms)	− 0.206	0.077
Anterior deltoid duration (ms)	− 0.352	0.002
Middle deltoid MVC (μV)	− 0.054	0.643
Middle deltoid amplitude (%MVC)	− 0.929	< 0.001
Middle deltoid onset (ms)	0.706	< 0.001
Middle deltoid offset (ms)	− 0.423	< 0.001
Middle deltoid duration (ms)	− 0.545	< 0.001
Posterior deltoid MVC (μV)	0.031	0.794
Posterior deltoid amplitude (%MVC)	− 0.889	< 0.001
Posterior deltoid onset (ms)	0.693	< 0.001
Posterior deltoid offset (ms)	− 0.547	< 0.001
Posterior deltoid duration (ms)	− 0.726	< 0.001
Pectoralis major MVC (μV)	− 0.753	< 0.001
Pectoralis major amplitude (%MVC)	− 0.388	< 0.001
Pectoralis major onset (ms)	0.411	< 0.001
Pectoralis major offset (ms)	− 0.754	< 0.001
Pectoralis major duration (ms)	− 0.761	< 0.001
Shoulder flexion ROM (degrees)	− 0.918	< 0.001
Shoulder abduction ROM (degrees)	− 0.913	< 0.001
Shoulder external rotation ROM (degrees)	− 0.857	< 0.001
Shoulder extension ROM (degrees)	− 0.872	< 0.001
Shoulder flexor strength (N)	− 0.943	< 0.001
Shoulder abductor strength (N)	− 0.943	< 0.001
Pain (0–10 scale)	0.642	< 0.001
DASH scores	0.831	< 0.001

Values are presented as *r* values which means very low (0 < *r* ≤ 0.19), low (0.2 ≤ *r* ≤ 0.39), moderate (0.4 ≤ *r* ≤ 0.59), high (0.6 ≤ *r* ≤ 0.79), and very high (0.8 ≤ *r* ≤ 0.19) correlation

### MANCOVA

MANCOVA controlling for weight and BMI revealed significant differences between lymphedema severity groups (Pillai’s Trace = 78.385, *F*(58, 86) = 78.385, *p* < 0.001, partial *η*2 = 0.981) for shoulder ROM, strength, function, and muscle activity (Table 4; full results in Supplementary file).

**Table 4:** MANCOVA Test results for between subject effects

Dependent variable	Mean square	*F*-value	*p* value	Partial Eta squared
Shoulder flexion ROM (degrees)	20,152.468	77.858	< 0.001	0.816
Shoulder abduction ROM (degrees)	18,751.058	74.522	< 0.001	0.810
Shoulder external rotation ROM (degrees)	6623.283	70.994	< 0.001	0.802
Shoulder extension ROM (degrees)	2757.848	65.354	< 0.001	0.789
Shoulder flexor strength (N)	68,827.687	269.732	< 0.001	0.939
Shoulder abductor strength (N)	54,375.830	211.496	< 0.001	0.924
DASH scores	7443.018	54.252	< 0.001	0.756
Biceps MVC (μV)	380,000.360	185.929	< 0.001	0.914
Biceps amplitude (%MVC)	1226.342	75.107	< 0.001	0.811
Biceps onset (ms)	8524.003	13.231	< 0.001	0.431
Biceps offset (ms)	294,538.615	263.518	< 0.001	0.938
Biceps duration (ms)	395,493.537	215.436	< 0.001	0.925
Anterior deltoid MVC (μV)	8383.917	0.899	0.469	0.049
Anterior deltoid amplitude (%MVC)	2480.701	125.999	< 0.001	0.878
Anterior deltoid onset (ms)	4186.507	77.474	< 0.001	0.816
Anterior deltoid offset (ms)	8032.127	1.763	0.146	0.092
Anterior deltoid duration (ms)	16,879.216	3.370	0.014	0.161
Middle deltoid MVC (μV)	1800.147	0.254	0.906	0.014
Middle deltoid amplitude (%MVC)	1282.041	144.953	< 0.001	0.892
Middle deltoid onset (ms)	1670.481	33.194	< 0	0.001
Middle deltoid offset (ms)	12,366.682	4.907	0.002	0.219
Middle deltoid duration (ms)	20,229.188	8.144	< 0.001	0.318
Posterior deltoid MVC (μV)	8680.340	0.768	0.549	0.042
Posterior deltoid amplitude (%MVC)	2168.182	118.277	< 0.001	0.871
Posterior deltoid onset (ms)	11,075.230	225.516	< 0.001	0.928
Posterior deltoid offset (ms)	8890.944	4.043	0.005	0.188
Posterior deltoid duration (ms)	38,609.770	17.028	< 0.001	0.493
Pectoralis major MVC (μV)	325,720.801	31.642	< 0.001	0.644
Pectoralis major amplitude (%MVC)	145.743	7.043	< 0.001	0.287
Pectoralis major onset (ms)	2993.033	6.176	< 0.001	0.261
Pectoralis major offset (ms)	213,758.212	32.129	< 0.001	0.647
Pectoralis major duration (ms)	265,670.500	33.120	< 0.001	0.654

## Discussion

This study demonstrated significant associations between BCRL severity and comprehensive shoulder dysfunction, showing progressive deterioration across mild, moderate, and severe groups. Multivariate analysis revealed substantial ROM and strength reductions (*p* < 0.001, partial *η*2 = 0.939), primarily in flexion and abduction. EMG analysis showed altered muscle activation patterns with reduced amplitudes and delayed onset times (*p* < 0.001), strongest in deltoids (*r* = − 0.938) and biceps (*r* = − 0.942). Higher DASH scores (64.92 ± 10.214) in severe cases confirmed the severity-dependent relationship between lymphedema and upper extremity function.

Shoulder ROM and strength reductions reflect lymphedema’s biomechanical impact. Severe cases showed marked limitations in flexion (73 ± 31°) and abduction (70 ± 29°), consistent with lymphatic fluid accumulation and tissue changes [46]. This aligns with lymphedema’s physiological cascade of fluid accumulation and fibrosis [47]. Decreased strength in flexors (31 ± 14N) and abductors (28 ± 13N) in severe cases demonstrates lymphedema’s impact on both joint mobility and force generation.

The EMG findings reveal complex neuromuscular adaptations associated with lymphedema severity. The significant reduction in EMG amplitudes across all muscle groups, particularly in the anterior deltoid (14.88 ± 5.819%MVC) and biceps (13.4 ± 3.916%MVC) in severe cases, suggests compromised muscle recruitment capabilities. Delayed muscle onset times suggest altered motor control, likely representing compensatory mechanisms due to lymphedema-induced mechanical constraints [48]. These changes reflect both direct tissue effects and adaptations to altered loads and proprioception.

The strong correlations between lymphedema severity and functional outcomes (DASH: *r* = 0.831, *p* < 0.001) demonstrate the progressive nature of upper extremity dysfunction. The relationship between severity and both objective measures (ROM, strength) and subjective outcomes (DASH) suggests a comprehensive impact on shoulder function. This multifaceted deterioration likely creates a cycle where reduced movement and altered muscle activation patterns may further compromise lymphatic drainage [49], potentially exacerbating lymphedema severity and resulting functional limitations.

The present study provides comprehensive quantitative evidence of the relationship between lymphedema severity and shoulder dysfunction of women with BCRL. Our findings both validate and extend previous research examining shoulder impairments in this population.

Baran et al. [8] used 3D motion analysis in 67 women, reporting decreased scapular upward rotation by 6.6° and posterior tilt by 3.6° in lymphedema cases compared to controls. They found significant reductions in shoulder abduction (165.3° vs 174.2°) and flexion (167° vs 172.6°) range of motion. Our results showed more dramatic mobility losses between mild and severe lymphedema groups in flexion (151° to 73°), abduction (144° to 70°), external rotation (76° to 33°), and extension (51° to 22°). While they focused on kinematics, our EMG analysis revealed preserved maximal contractions (1200 μV) but impaired recruitment timing (anterior deltoid: − 20 to 10 ms; middle deltoid: − 10 to 10 ms) and amplitude (anterior deltoid: 42.76% to 14.88%MVC; middle deltoid: 42.52 to 23.04%MVC) in severe cases.

Korucu et al. [50] compared 50 lymphedema versus 57 non-lymphedema patients, finding higher rates of scapular dyskinesia (70% vs 59.6%) and reduced pain thresholds in trapezius (15.35N vs 12.43N) and deltoid (13.30N vs 11.80N) muscles. They reported DASH scores of 35.83 for lymphedema versus 26.66 for non-lymphedema groups. Our study demonstrated more severe functional decline with DASH scores progressively increasing from mild (17.8) to moderate (32.0) to severe (64.92) lymphedema. Our EMG analysis further quantified the neuromuscular basis for these functional deficits, showing progressive reductions in muscle activation amplitudes (anterior deltoid: 42.76 to 14.88%MVC) and delayed onset timing (anterior deltoid: − 20 to 10 ms) that scaled with increasing lymphedema severity.

Hayes et al. [12] longitudinal study of 2442 women showed that heaviness and tightness symptoms strongly predicted lymphedema development (OR:1.94). They found approximately two in three women reported > 2 symptoms of mild severity, while one in three reported moderate severity symptoms. The presence of symptoms was associated with poorer upper-body function and lower physical activity levels. Our EMG findings provide the neuromuscular mechanism underlying these symptoms, revealing progressive losses in muscle activation from mild to severe lymphedema in anterior deltoid (42.76 to 14.88%MVC), middle deltoid (42.52 to 23.04%MVC), and posterior deltoid (37.96 to 13.16%MVC). We also found significant timing deficits with onset delays increasing from − 20 to 10 ms, matching their observation that symptoms worsen with severity.

Prieto-Gómez et al. [51] EMG study of 90 women found delayed serratus anterior activation (2.08 s vs 1.09 s) and reduced amplitude (4.75% vs 22.63%MVC) in breast cancer patients versus controls. We similarly demonstrated timing deficits across multiple shoulder muscles, with anterior deltoid (− 20 to 10 ms), middle deltoid (− 10 to 10 ms), posterior deltoid (− 40 to 10 ms), biceps (26.2 to 72.6 ms), and pectoralis major (24.4 to 51.2 ms) showing progressive delays with lymphedema severity. However, unlike their findings, we observed preserved maximal voluntary contraction amplitudes across severity levels in the deltoid muscles (1200 μV), suggesting potential for targeted rehabilitation.

Our results demonstrate that shoulder dysfunction in breast cancer–related lymphedema follows a clear severity-dependent pattern. The EMG findings of delayed muscle onset timing and reduced activation amplitudes, while maintaining maximal force capacity, provide a strong rationale for early intervention. Clinicians should focus on restoring normal muscle recruitment patterns, particularly in the deltoid and pectoralis major muscles where we observed the most significant timing deficits. The severe reductions in shoulder mobility (> 50% in severe cases) and strength emphasize the necessity of ROM and progressive resistance exercises. However, the strong correlation between lymphedema severity and functional decline (DASH scores 17.8 to 64.9) underscores the need for carefully prescribed exercise with appropriate compression to prevent symptom exacerbation.

Future research should examine longitudinal changes in shoulder muscle activation patterns as lymphedema progresses, incorporating fine-wire EMG for deep rotator cuff analysis. Randomized controlled trials evaluating targeted neuromuscular interventions based on severity level are needed. Investigation of motor unit characteristics and dynamic shoulder function during functional tasks would enhance clinical translation.

## Strength and limitations

Strengths include comprehensive EMG analysis of multiple shoulder muscles using precise electrode placement and standardized protocols, robust quantification of muscle activation patterns, and synchronous assessment of timing/amplitude characteristics. The multi-modal assessment approach combining EMG, strength testing, ROM measurements, and functional outcomes provided rich data integration. EMG signal processing followed established guidelines with excellent reliability coefficients.

Study limitations include the cross-sectional design, which only establishes associations rather than causal relationships between lymphedema severity and shoulder dysfunction. Surface EMG restricted analysis to superficial muscles, excluding deeper rotator cuff assessment. The single testing session and isometric-only strength measurements may not fully represent functional abilities. Additional EMG parameters like frequency content and motor unit firing patterns could provide deeper neuromuscular insights. Longitudinal studies with dynamic assessments are needed to confirm these findings.

## Conclusion

This study found that women with severe lymphedema exhibited substantially reduced shoulder strength, range of motion, and scapular mobility along with increased pain and upper limb disability compared to those with milder forms; surface electromyography also revealed impaired neuromuscular activation patterns that may underlie declining function.

## Supplementary Information

Below is the link to the electronic supplementary material.

Supplementary file1 (DOCX 28 KB)

## Data Availability

No datasets were generated or analysed during the current study.
