# Correction to: The impact of lymphedema severity on shoulder joint function and muscle activation patterns in breast cancer survivors: a cross‑sectional study

**DOI:** 10.1007/s00520-025-10199-0

**Published:** 2025-11-21

**Authors:** Mahmoud Hamada Mohamed, Rafik E. Radwan, Mohamed M. ElMeligie, Abdelrazak Ahmed, Hend R. Sakr, Mahmoud ElShazly

**Affiliations:** 1 Department of Physical Therapy for Surgery, Faculty of Physical Therapy, Ahram Canadian University, 4 Industrial Zone, Banks Complex, 6th of October City, Giza, Egypt; 2 Department of Biomechanics, Faculty of Physical Therapy, Cairo University, Cairo, Egypt; 3 Department of Basic Sciences, Faculty of Physical Therapy, Ahram Canadian University, Giza, Egypt; 4 Department of Physical Therapy for Neuromuscular Disorders and Its Surgery, Faculty of Physical Therapy, South Valley University, Qena, Egypt; 5 Lecturer of Physical Therapy, Department of Women’s Health, Faculty of Physical Therapy, Badr University, Badr, Egypt; 6 Department of Physical Therapy for Surgery, Faculty of Physical Therapy, South Valley University, Qena, Egypt

**Correction to: Supportive Care in Cancer (2025) 33:37**

10.1007/s00520-024-09044-7

Upon reviewing the published version of our article titled "The impact of lymphedema severity on shoulder joint function and muscle activation patterns in breast cancer survivors: a cross-sectional study", we identified an authorial administrative error in the Ethics approval statement. The article currently lists the approving body as the Research Ethics Committee, Faculty of Physical Therapy, Cairo University with reference 010/28202306. This is unfortunately incorrect.

The correct approving body is the Research Ethics Committee, Faculty of Physical Therapy, South Valley University, Egypt, Protocol ID: P.T-SUR-08/2023–517.

The original article has been corrected.

