# Supplementary material for: The impact of lymphedema severity on shoulder joint function and muscle activation patterns in breast cancer survivors: a cross-sectional study
Source: Support Care Cancer. 2024 Dec 17;33(1):37. doi: 10.1007/s00520-024-09044-7 (PMC11652595; doi:10.1007/s00520-024-09044-7)
Supplement: Supplementary file 1 — Supplementary file1 (DOCX 28 KB) [file 520_2024_9044_MOESM1_ESM.docx]

1. **MANCOVA Multivariate Tests**

| Effect | Value | F | df1 | df2 | Sig. | Partial Eta Squared |
| --- | --- | --- | --- | --- | --- | --- |
| Intercept | Pillai's Trace | 42.881 | 29 | 42 | <0.001 | 0.967 |
| Weight | Pillai's Trace | 1.222 | 29 | 42 | 0.272 | 0.458 |
| BMI | Pillai's Trace | 0.763 | 29 | 42 | 0.776 | 0.345 |
| Group | Pillai's Trace | 78.385 | 58 | 86 | <0.001 | 0.981 |

1. **Tests of Between-Subjects Effects**

| Dependent Variable | Mean Square | F | Sig. | Partial Eta Squared |
| --- | --- | --- | --- | --- |
| Shoulder Flexion ROM (degrees) | 20152.468 | 77.858 | <0.001 | 0.816 |
| Shoulder Abduction ROM (degrees) | 18751.058 | 74.522 | <0.001 | 0.810 |
| Shoulder External Rotation ROM (degrees) | 6623.283 | 70.994 | <0.001 | 0.802 |
| Shoulder Extension ROM (degrees) | 2757.848 | 65.354 | <0.001 | 0.789 |
| Shoulder Flexor Strength (N) | 68827.687 | 269.732 | <0.001 | 0.939 |
| Shoulder Abductor Strength (N) | 54375.830 | 211.496 | <0.001 | 0.924 |
| DASH scores | 7443.018 | 54.252 | <0.001 | 0.756 |
| Biceps MVC (μV) | 380000.360 | 185.929 | <0.001 | 0.914 |
| Biceps Amplitude (%MVC) | 1226.342 | 75.107 | <0.001 | 0.811 |
| Biceps Onset (ms) | 8524.003 | 13.231 | <0.001 | 0.431 |
| Biceps Offset (ms) | 294538.615 | 263.518 | <0.001 | 0.938 |
| Biceps Duration (ms) | 395493.537 | 215.436 | <0.001 | 0.925 |
| Anterior Deltoid MVC (μV) | 8383.917 | 0.899 | 0.469 | 0.049 |
| Anterior Deltoid Amplitude (%MVC) | 2480.701 | 125.999 | <0.001 | 0.878 |
| Anterior Deltoid Onset (ms) | 4186.507 | 77.474 | <0.001 | 0.816 |
| Anterior Deltoid Offset (ms) | 8032.127 | 1.763 | 0.146 | 0.092 |
| Anterior Deltoid Duration (ms) | 16879.216 | 3.370 | 0.014 | 0.161 |
| Middle Deltoid MVC (μV) | 1800.147 | 0.254 | 0.906 | 0.014 |
| Middle Deltoid Amplitude (%MVC) | 1282.041 | 144.953 | <0.001 | 0.892 |
| Middle Deltoid Onset (ms) | 1670.481 | 33.194 | <0 | 0.001 |
| Middle Deltoid Offset (ms) | 12366.682 | 4.907 | 0.002 | 0.219 |
| Middle Deltoid Duration (ms) | 20229.188 | 8.144 | <0.001 | 0.318 |
| Posterior Deltoid MVC (μV) | 8680.340 | 0.768 | 0.549 | 0.042 |
| Posterior Deltoid Amplitude (%MVC) | 2168.182 | 118.277 | <0.001 | 0.871 |
| Posterior Deltoid Onset (ms) | 11075.230 | 225.516 | <0.001 | 0.928 |
| Posterior Deltoid Offset (ms) | 8890.944 | 4.043 | 0.005 | 0.188 |
| Posterior Deltoid Duration (ms) | 38609.770 | 17.028 | <0.001 | 0.493 |
| Pectoralis Major MVC (μV) | 325720.801 | 31.642 | <0.001 | 0.644 |
| Pectoralis Major Amplitude (%MVC) | 145.743 | 7.043 | <0.001 | 0.287 |
| Pectoralis Major Onset (ms) | 2993.033 | 6.176 | <0.001 | 0.261 |
| Pectoralis Major Offset (ms) | 213758.212 | 32.129 | <0.001 | 0.647 |
| Pectoralis Major Duration (ms) | 265670.500 | 33.120 | <0.001 | 0.654 |

1. **Estimated Marginal Means**

| Dependent Variable | Group 1 Mean (SE) | Group 2 Mean (SE) | Group 3 Mean (SE) |
| --- | --- | --- | --- |
| Shoulder Flexion ROM (degrees) | 148.899 (4.610) | 115.534 (3.249) | 72.888 (4.527) |
| Shoulder Abduction ROM (degrees) | 142.990 (4.545) | 110.650 (3.203) | 69.920 (4.464) |
| Shoulder External Rotation ROM (degrees) | 74.916 (2.767) | 45.680 (1.951) | 33.124 (2.718) |
| Shoulder Extension ROM (degrees) | 50.133 (1.861) | 39.932 (1.312) | 22.015 (1.828) |
| Shoulder Flexor Strength (N) | 173.108 (4.577) | 79.896 (3.226) | 33.596 (4.495) |
| Shoulder Abductor Strength (N) | 157.948 (4.594) | 94.032 (3.238) | 29.740 (4.512) |
| DASH scores | 13.004 (3.356) | 32.119 (2.365) | 69.597 (3.296) |
| Biceps MVC (μV) | 771.240 (12.953) | 629.235 (9.129) | 437.525 (12.722) |
| Biceps Amplitude (%MVC) | 33.918 (1.158) | 23.296 (0.816) | 12.746 (1.137) |
| Anterior Deltoid MVC (μV) | 1208.207 (27.666) | 1176.892 (19.499) | 1130.901 (27.173) |
| Dependent Variable | Group 1 Mean (SE) | Group 2 Mean (SE) | Group 3 Mean (SE) |
| Shoulder Flexion ROM (degrees) | 148.899 (4.610) | 115.534 (3.249) | 72.888 (4.527) |
| Shoulder Abduction ROM (degrees) | 142.990 (4.545) | 110.650 (3.203) | 69.920 (4.464) |
| Shoulder External Rotation ROM (degrees) | 74.916 (2.767) | 45.680 (1.951) | 33.124 (2.718) |
| Shoulder Extension ROM (degrees) | 50.133 (1.861) | 39.932 (1.312) | 22.015 (1.828) |
| Shoulder Flexor Strength (N) | 173.108 (4.577) | 79.896 (3.226) | 33.596 (4.495) |
| Shoulder Abductor Strength (N) | 157.948 (4.594) | 94.032 (3.238) | 29.740 (4.512) |
| DASH scores | 13.004 (3.356) | 32.119 (2.365) | 69.597 (3.296) |
| Biceps MVC (μV) | 771.240 (12.953) | 629.235 (9.129) | 437.525 (12.722) |
| Biceps Amplitude (%MVC) | 33.918 (1.158) | 23.296 (0.816) | 12.746 (1.137) |
| Anterior Deltoid MVC (μV) | 1208.207 (27.666) | 1176.892 (19.499) | 1130.901 (27.173) |
| Anterior Deltoid Amplitude (%MVC) | 42.802 (1.271) | 32.277 (0.896) | 14.881 (1.249) |
| Anterior Deltoid Onset (ms) | -17.591 (2.106) | 10.108 (1.484) | 9.083 (2.069) |
| Anterior Deltoid Offset (ms) | 961.197 (19.341) | 926.265 (13.632) | 877.138 (18.996) |
| Anterior Deltoid Duration (ms) | 978.788 (20.278) | 916.157 (14.292) | 868.055 (19.917) |
| Middle Deltoid MVC (μV) | 1194.991 (24.132) | 1191.085 (17.009) | 1201.924 (23.703) |
| Middle Deltoid Amplitude (%MVC) | 43.260 (0.852) | 34.619 (0.601) | 23.040 (0.837) |
| Middle Deltoid Onset (ms) | -9.850 (2.033) | 9.183 (1.433) | 9.467 (1.996) |
| Middle Deltoid Offset (ms) | 936.145 (14.383) | 915.592 (10.137) | 862.263 (14.127) |
| Middle Deltoid Duration (ms) | 946.010 (14.279) | 906.422 (10.064) | 852.968 (14.025) |
| Posterior Deltoid MVC (μV) | 1068.181 (30.452) | 1060.869 (21.463) | 1058.949 (29.910) |
| Posterior Deltoid Amplitude (%MVC) | 37.923 (1.227) | 33.276 (0.865) | 13.162 (1.205) |
| Posterior Deltoid Onset (ms) | -42.963 (2.008) | 10.792 (1.415) | 11.571 (1.972) |
| Posterior Deltoid Offset (ms) | 809.450 (13.436) | 774.417 (9.470) | 759.932 (13.197) |
| Posterior Deltoid Duration (ms) | 851.905 (13.643) | 763.714 (9.616) | 747.981 (13.400) |
| Pectoralis Major MVC (μV) | 883.229 (29.069) | 788.728 (20.488) | 621.243 (28.552) |
| Pectoralis Major Amplitude (%MVC) | 28.591 (1.303) | 25.324 (0.919) | 21.325 (1.280) |
| Pectoralis Major Onset (ms) | 22.255 (6.307) | 36.686 (4.445) | 51.858 (6.195) |
| Pectoralis Major Offset (ms) | 710.869 (23.370) | 631.866 (16.472) | 495.865 (22.954) |
| Pectoralis Major Duration (ms) | 688.613 (25.661) | 595.180 (18.086) | 444.007 (25.204) |
